# Morphological and Glucose Metabolism Abnormalities in Alcoholic Korsakoff's Syndrome: Group Comparisons and Individual Analyses

**DOI:** 10.1371/journal.pone.0007748

**Published:** 2009-11-13

**Authors:** Anne-Lise Pitel, Anne-Marie Aupée, Gaël Chételat, Florence Mézenge, Hélène Beaunieux, Vincent de la Sayette, Fausto Viader, Jean-Claude Baron, Francis Eustache, Béatrice Desgranges

**Affiliations:** 1 Inserm – EPHE – Université de Caen/Basse-Normandie, Unité U923, GIP Cyceron, CHU Côte de Nacre, Caen, France; 2 Department of Psychiatry and Behavioral Sciences, Stanford University, Stanford, California, United States of America; 3 CHU Cote de Nacre, Département de Neurologie, Caen, France; 4 University of Cambridge, Department of Clinical Neurosciences, Neurology Unit, Cambridge, United Kingdom; University of Granada, Spain

## Abstract

**Background:**

Gray matter volume studies have been limited to few brain regions of interest, and white matter and glucose metabolism have received limited research attention in Korsakoff's syndrome (KS). Because of the lack of brain biomarkers, KS was found to be underdiagnosed in postmortem studies.

**Methodology/Principal Findings:**

Nine consecutively selected patients with KS and 22 matched controls underwent both structural magnetic resonance imaging and ^18^F-fluorodeoxyglucose positron emission tomography examinations. Using a whole-brain analysis, the between-group comparisons of gray matter and white matter density and relative glucose uptake between patients with KS and controls showed the involvement of both the frontocerebellar and the Papez circuits, including morphological abnormalities in their nodes and connection tracts and probably resulting hypometabolism. The direct comparison of the regional distribution and degree of gray matter hypodensity and hypometabolism within the KS group indicated very consistent gray matter distribution of both abnormalities, with a single area of significant difference in the middle cingulate cortex showing greater hypometabolism than hypodensity. Finally, the analysis of the variability in the individual patterns of brain abnormalities within our sample of KS patients revealed that the middle cingulate cortex was the only brain region showing significant GM hypodensity and hypometabolism in each of our 9 KS patients.

**Conclusions/Significance:**

These results indicate widespread brain abnormalities in KS including both gray and white matter damage mainly involving two brain networks, namely, the fronto-cerebellar circuit and the Papez circuit. Furthermore, our findings suggest that the middle cingulate cortex may play a key role in the pathophysiology of KS and could be considered as a potential in vivo brain biomarker.

## Introduction

Korsakoff's syndrome (KS) is marked by global amnesia, which develops either insidiously or in the wake of Wernicke's encephalopathy and whose most common etiology is the combination of thiamine deficiency and alcoholism [1]. In addition to severe anterograde amnesia [2], alcoholic Korsakoff's syndrome encompasses other neuropsychological impairments, such as executive dysfunctions [3], retrograde amnesia [1], visuospatial deficits [4] and ataxia of gait and balance [5].

Postmortem studies of alcoholic Korsakoff patients have shown pathological abnormalities involving periventricular and periaqueductal gray matter, walls of the third ventricle, floor of the fourth ventricle and cerebellum [6]. Damage has also been found in the hippocampus [7], certain nuclei of the thalamus [8], [9], hypothalamus [10] and more particularly the mammillary bodies [11], [12], cerebral cortex [13], brainstem nuclei [14] and locus coeruleus [15]. Neuroimaging studies using computerized tomography have revealed morphological abnormalities, involving cortical volume reduction, ventricular enlargement, Sylvian fissure and frontal sulcus widening, wider interhemispheric fissure and thalamic hypodensity [4], [16]. More specifically, magnetic resonance imaging (MRI) has highlighted decreased volume of the parietal [17] and frontal cortex [17]–[20], thalamus [3], [18], [20]–[23] and mammillary bodies [3], [20], [23]–[25]. Findings in the medial temporal lobe of patients with KS are more controversial, with the hippocampus being reported as either preserved [18], [26] or damaged [22], [23], [27].

These previous MRI investigations, based on the region of interest (ROI) method, have provided considerable and robust insight into morphological abnormalities characterizing KS. However, because they were hypothesis-driven, they only assessed a fraction of the brain parenchyma and may have missed abnormalities in regions of the brain which were not examined. Moreover, very few in vivo studies have examined white matter in this pathology [17], [21], [28], even though reductions in white matter volume have been found in neuropathological studies of KS [13], [29], and chronic alcoholism is known to affect white matter macrostructure [30] and microstructure [31], [32]. A voxel-based (voxel-based morphometry, VBM) examination of both gray and white matter damage in KS would thus be useful for providing a comprehensive assessment of the morphological brain alterations characterizing this syndrome.

Brain abnormalities may also be functional, sometimes involving remote structures connected to the area of primary damage. In contrast to structural MRI, positron emission tomography (PET) represents a means of assessing the dysfunction of neural networks through measurements of resting cerebral blood flow or glucose metabolic rate (CMRGlc), which are closely related to synaptic activity [33]. Using resting fluorodeoxyglucose (FDG) PET methodology, decreased relative CMRGlc in the anterior and posterior cingulate cortex and in the precuneus (with a trend for the thalamus) has already been observed in KS [34]. In another FDG-PET study [35], the inferior and middle frontal lobes were also found to be involved, as well as the parietal and orbitofrontal cortices, though with marginal statistical significance. In this study, however, FDG uptake was measured during a recognition task, which may have obscured the hypometabolism pattern by increasing activity in some cerebral structures relative to the resting state. In addition, both studies employed the ROI approach, which provides only limited insight into metabolic brain abnormalities. Two PET studies have used voxel-based analysis of FDG-PET in KS [20], [36]. In three KS patients, Aupée et al. (2001) highlighted the presence of hypometabolism in the thalamus, posterior cingulate and mesial frontal cortices, and left supramarginal and middle temporal gyri. More recently, Reed et al. (2003) reported significant hypometabolism in the thalamus, medial temporal lobe and retrosplenial cortex in 12 KS. However, these two voxel-based studies used low-resolution PET devices ([36]: 5.5×5.5×9 mm; [20]: 8.8×8.5×5.5 mm), with a restricted axial field of view ([36]: 81 mm; [20]: 108 mm), which may have missed hypometabolic foci. Metabolic abnormalities in KS would be localized more accurately with a voxel-based analysis of data acquired with a high-resolution scanner and a wider axial field of view.

Although two previous studies have investigated KS by combining MRI with either single photon emission computed tomography [37] or PET [20], neither was designed to compare the location and severity of morphological versus metabolism abnormalities. And yet, such a direct comparison might offer evidence for local discrepancies or concordance between morphological and metabolism abnormalities, as reported in Alzheimer's disease [38] and semantic dementia [39] respectively.

Lastly, the existence of invariability in the individual patterns of brain abnormalities in KS, which could be regarded as a marker of this disease, is still a matter of debate. Most imaging investigations have involved group comparisons between KS and controls, and only a few have examined individual patterns of brain abnormalities. Previous neuropathological and structural imaging investigations have provided conflicting results regarding the role of the mammillary bodies [40], [41] and medio-dorsal thalamus [9], [40], reported by some to be invariably damaged in patients with KS and by others to be preserved in certain patients. Likewise, glucose hypometabolism was invariably found in a group of three KS patients, in the bilateral posterior cingulate and thalamus, left supramarginal gyrus and bilateral mesial frontal cortex [36]. Thus, although KS appears to result from the disruption of the Papez hippocampo-mammillothalamic circuit, an invariant morphological and metabolism pattern of abnormalities remains elusive.

The goals of the present study were threefold. Firstly, we aimed to provide a comprehensive assessment of gray matter (GM) and white matter (WM) density, as well as glucose metabolism, in a group of KS patients, using voxel-based analyses. Secondly, we wanted to compare the distributions and degrees of morphological and metabolism GM abnormalities using a method specially designed for this purpose. As hypodensity and hypometabolism do not have the same units and normative values, we therefore computed, for each patient, the MRI and PET Z-score maps relative to normative data obtained from the same sample of control subjects, and then compared the Z-score maps between the two modalities. The third goal was to determine a potential in vivo brain biomarker of KS. To this end, we examined the frequency of the patterns of morphological and metabolism abnormalities in the KS sample by using a novel way to use and display the results of the individual z-score maps.

## Results

### Between-Group Comparisons

**Figure 1:**
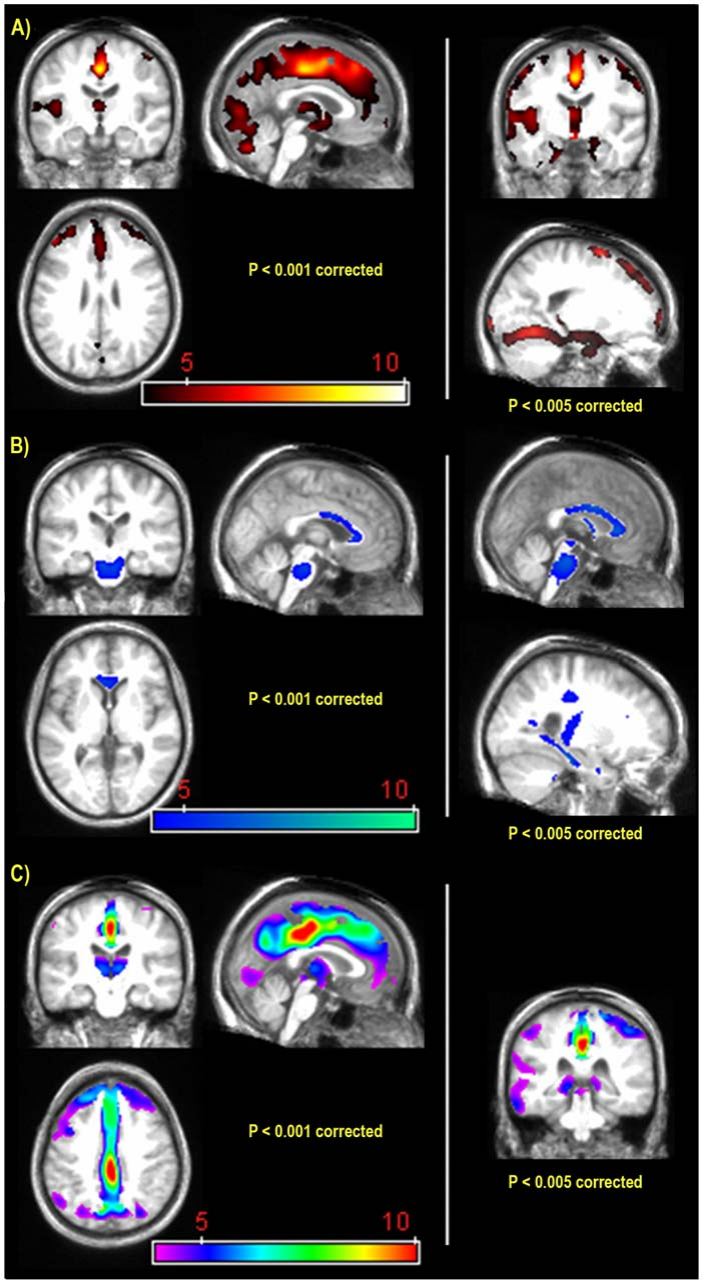
GM (A), WM (B), and metabolism (C) abnormalities in Korsakoff's syndrome compared with healthy subjects (k>200 voxels). Between group comparisons conducted with the SPM5 two-sample t-test routine. False Discovery Rate correction. On the coronal slice, the right is displayed on the right. The colored bar represents the t value of significant voxels.

As illustrated in Figure 1A, the analysis revealed significantly (*p*<0.001) lower GM density in the KS group than controls bilaterally in the cerebellum, lingual gyrus, fusiform gyrus, dorsomedian thalamus, hypothalamus (particularly the mammillary bodies), median and superior orbitofrontal cortex, superior and middle frontal cortex, supplementary motor area, anterior and middle cingulate cortex, cuneus, precuneus and paracentral lobule, left superior temporal gyrus, Heschl's gyrus, insula, supramarginal gyrus, and right postcentral and precentral gyri. When a less stringent FDR-corrected *p*-value cut-off of <0.005 was applied, the bilateral hippocampal, parahippocampal and posterior cingulate cortices were also identified with lower GM density in KS than controls.

Regarding WM density, significant (*p*<0.001) decreases involved the genu and body of the corpus callosum, the cerebellar WM and the pontine and mesencephalic WM in the KS group compared with controls (Figure 1B). Using a less stringent FDR-corrected *p*-value cut-off of <0.005, the superior part of the fornix and the inferior part of the cingulum bundle were also implicated bilaterally.

Significant hypometabolism in KS was present bilaterally and involved the calcarine cortex, lingual gyrus, middle occipital gyrus, thalamus, mammillary bodies, median orbitofrontal cortex, superior middle frontal cortex, supplementary motor area, whole cingulate cortex, cuneus, precuneus and paracentral lobule, as well as the left middle and inferior temporal lobe. Using a less stringent FDR-corrected *p*-value cut-off of <0.005, significant abnormalities were also observed in the bilateral hippocampal and left parahippocampal areas (Figure 1C).

### Within-Group Analysis

**Figure 2:**
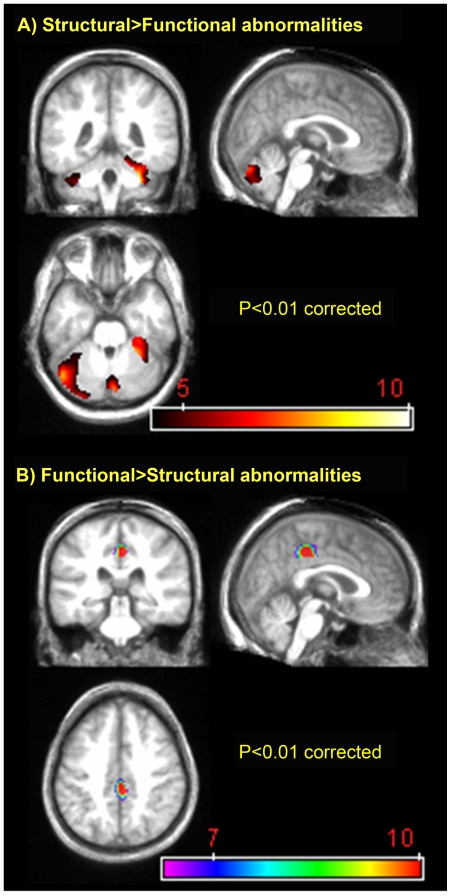
Voxel-based comparison between morphological and metabolism GM abnormalities in Korsakoff's syndrome (*p*<0.01 FDR; k>200 voxels). Within group comparisons conducted with the SPM5 paired t-test routine, using z-score maps for hypodensity and hypometabolism. False Discovery Rate correction. On the coronal slice, the right is displayed on the right. The colored bar represents the t value of significant voxels.

The direct SPM comparison between MRI and PET z-scores (including only voxels with group mean Z-MRI and/or Z-PET <−1.725) is illustrated in Figure 2. The GM hypodensity>hypometabolism contrast highlighted the cerebellum and fusiform gyrus bilaterally (Figure 2A), while the reverse GM hypometabolism>hypodensity contrast revealed a single cluster in the middle cingulate cortex (Figure 2B).

### Individual Patterns

**Figure 3:**
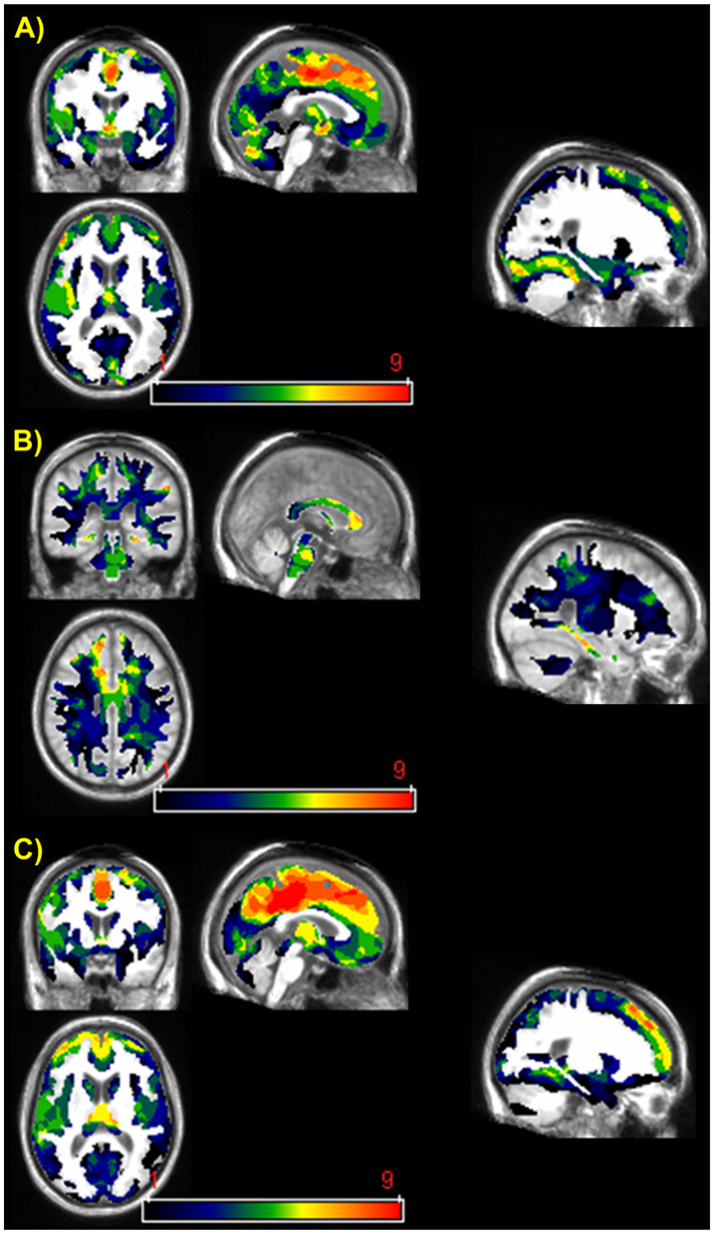
Variability in the individual patterns of GM (A) and WM (B) hypodensity, and GM hypometabolism (C) in the patients with Korsakoff's syndrome. The colored bar represents the variability of the brain abnormalities across the KS sample from present (red) in all 9 KS patients to absent (black) in all 9 KS.

The middle cingulate gyrus was the only structure showing significant GM hypodensity in all 9 KS patients (Figure 3A). No consistent WM abnormality was found (Figure 3B), while consistent hypometabolism was found to involve the middle cingulate gyrus, precuneus and superior frontal gyrus in all 9 KS patients (Figure 3C).

## Discussion

This study revealed widespread morphological and metabolism abnormalities in KS patients, but two networks appeared to be particularly impaired, namely the frontocerebellar and Papez circuits. Moreover, the middle cingulate cortex, which belongs to the Papez circuit, seemed to play a key role in the neuropathology of KS.

### Frontocerebellar Circuit

Our findings are in general agreement with previous studies reporting GM abnormalities in the frontal lobes [17], [20], cerebellum [5], [41] and thalamus [18], [20], [23] and glucose hypometabolism in the frontal lobes and thalamus [20], [36]. Our study also revealed WM hypodensity in cerebellar, pontine and mesencephalic WM. Taken together, these findings suggest changes to the main nodes and connections of the frontocerebellar circuitry linking the frontal cortex to the thalamus and cerebellum via the ventral pons [42], [43]. Disruption of this circuit may be related, or lead to, both the gait and balance deficits [5] and some of the cognitive impairments (executive functions and working memory) observed in KS [2].

A comparison of morphological and metabolism abnormalities showed that GM hypodensity was significantly greater than hypometabolism in the cerebellar hemispheres, suggesting the relative metabolic preservation of these structures. This finding is in agreement with a previous report indicating that the severity of morphological abnormalities does not correlate with local metabolic rates in the cerebellum in uncomplicated alcoholism [44], possibly reflecting compensatory mechanisms within the frontocerebellar circuit [45]. Our findings in the cerebellum need, however, to be interpreted with caution, as the fact we used the vermis, a region adjoining the cerebellar hemispheres, as reference for the quantitative normalization of PET data may have influenced these results.

Lastly, abnormalities in the pontine and mesencephalic fibers were suggestive of the presence of a subclinical form of central pontine myelinolysis in KS, as has already been noted in uncomplicated alcoholism [46]. The same hypothesis may be established regarding the corpus callosum and the existence of a subclinical form of Marchiafava-Bignami disease in KS [47].

### Papez Circuit

We found significantly lower GM density in the thalamus and hypothalamus (particularly affecting the mammillary bodies) of KS than controls, which is in agreement with neuropathological data [9], [13], [48], [49] and previous MRI investigations using the ROI approach [18], [20], [23], [41], [50]. We also found hippocampal and parahippocampal GM hypodensity, confirming the presence of medial temporal abnormalities in KS [22], [23], [27]. In addition to morphological abnormalities in the nodes of the Papez circuit, our study revealed disruption of connectivity, involving the superior part of the fornix and inferior part of the cingulum bundle, leading to disconnections between the cingulate gyri, hippocampi and mammillary bodies.

The morphological damage to the nodes and connections of the Papez circuit may have caused synaptic dysfunction, as revealed by the hypometabolism affecting the thalamus, mammillary bodies, anterior cingulate cortex, medial temporal lobe and, more precisely, the hippocampus and left parahippocampal cortex [20], [34]–[36]. This network dysfunction may in turn account for the severe episodic memory disorders in KS [2], [51]. Further studies using diffusion tensor imaging may reveal a precise pattern in the fiber bundle damage and circuit disconnection and allow links to be drawn between microstructural GM and WM changes and episodic memory impairment in this syndrome.

Lastly, our individual analysis confirms the findings of a number of previous post-mortem and in vivo imaging studies indicating that neither morphological [41] nor metabolism [36] abnormalities in the thalamus or the mammillary bodies can be regarded as brain markers of KS.

### Middle Cingulate Cortex

The voxel-by-voxel analysis revealed that the morphological and metabolism abnormalities were most severe in the cingulate cortex, especially its middle part. The cingulate cortex has already been shown to be heavily involved in other memory disorders, including Alzheimer disease [38], [52]. To our knowledge, the present study is the first to report morphological damage in this structure in KS, although glucose hypometabolism there has been already reported [34]–[36]. Our findings, showing both morphological and metabolism abnormalities in the middle cingulate cortex, challenge the hypothesis that the retrosplenial hypometabolism in KS is entirely secondary to morphological damage within the diencephalic-limbic memory circuits [20].

However, we also showed that the middle cingulate cortex was the only structure to exhibit greater hypometabolism than GM hypodensity. This suggests that hypometabolism in this structure may not only be due to local neuronal damage but may also reflect synaptic dysfunction remote from morphological damage in other components of the Papez circuit. Hypometabolism in the cingulate cortex may, in fact, have preceded the morphological damage there, and may be related to the harmful effects of alcohol on neurotransmission [53] and WM integrity [54] within the Papez circuit.

The final aim of the present study was to analyze the variability of the morphological and metabolism brain abnormalities in KS. The middle cingulate cortex was the only structure to exhibit both GM hypodensity and hypometabolism, suggesting that KS, which is regarded as the prototype of diencephalic amnesia, may be characterized by this systematic involvement of the cingulate cortex. Indeed, the middle cingulate cortex may play a key role in the pathophysiology of KS, given that 1) it was the brain area most severely affected both morphologically and metabolically, 2) it was the only brain area to exhibit greater hypometabolism than GM hypodensity, and 3) it was consistently damaged across the entire KS sample. The middle cingulate cortex may therefore be considered as a potential brain biomarker of Korsakoff syndrome, which would facilitate the in vivo detection of this underdiagnosed neurological disorder using clinical imaging techniques [11], [55], [56].

### Limitations

This study has certain limitations. Firstly, the present conclusions are based on a relatively small sample of patients, as it is challenging to collect a larger group of carefully selected KS patients with both MRI and PET data. Secondly, we used the MNI template for registration (spatial normalization) and segmentation instead of a customized template from our samples of controls and KS patients. While this is the recommended procedure for samples of less than hundreds of subjects, this may however lead to reduce the accuracy of the registration process, particularly for KS patients. We thus checked the accuracy of our registration process firstly by checking how each individual registered image matched with the MNI template and each other, and secondly, by overlaying our results on the mean of the controls' and KS patients' registered images as well as on each individual KS patients' registered image. Finally, even though the present study is the first to highlight the potential existence of an in vivo brain biomarker in KS, it doesn't allow us to specify whether the observed brain abnormalities are related to thiamine deficiency or chronic alcoholism. Indeed, some of the above-mentioned morphological and functional abnormalities have also been described in uncomplicated alcoholism, suggesting graded effects of brain abnormalities [47]. Further studies, including a group of uncomplicated alcoholic controls without vitamin depletion, are required to specify the roles of these two factors.

## Materials and Methods

### Subjects

Nine patients with KS (5 men and 4 women, age 57.4±11.1) and 22 age- and sex-matched controls (9 men and 13 women, age 60.1±4.5) were enrolled. None of the participants presented history of psychiatric or neurological problems (head injury, coma, epilepsy, depression, etc.). The participants gave their written informed consent prior to their inclusion in the study, which was conducted in line with the Declaration of Helsinki and was approved by the local ethical committee (Comité Consultatif de Protection des Personnes en Recherche Biomédicale de Basse-Normandie), allowing this study to be conducted in Cyceron imaging center and Caen University Hospital (CHU).

The KS patients were diagnosed with reference to the DSM IV [57] criteria of “Persisting Amnestic Disorder” and all met criteria by chart review for Wernicke's encephalopathy [58]. Even though it was difficult to gain an accurate picture of their lifetime alcohol intake because of the amnesia, they all had a history of heavy drinking as reported by family members and medical records. Each patient underwent a detailed neuropsychological examination comprising assessments of episodic memory, intelligence or abstract reasoning, and executive functions. Episodic memory was assessed by means of one or more of the following tests: the Signoret Memory Battery [59], Rey-Osterrieth Complex Figure copy and recall [60], the Wechsler Memory Scale [61], the Grober and Buschke test [62], and the California Verbal Learning Test [63], which evaluate verbal and visual memory in immediate and delayed recall. Intelligence or abstract reasoning was assessed by means of Raven's Progressive Matrices [64] or by an intelligence quotient (Beauregard's IQ, [65]). Lastly, executive functions were measured using a verbal fluency test [66], the Stroop test [67] or the Wisconsin Card Sorting Test [68]. All the patients presented disproportionately severe episodic memory disorders compared with other cognitive functions and their memory impairments had social repercussions, in accordance with DSM IV criteria. Indeed, none of the KS were able to go back to their previous jobs and all of them lived in sheltered accommodation or were inpatients waiting for a place in an institution.

Controls were social drinkers as defined by the National Institute on Alcohol Abuse and Alcoholism [69].

### Imaging Data Acquisition

#### MRI

For each subject, a high-resolution T1-weighted volume MRI scan was obtained, which consisted of a set of 128 adjacent axial slices parallel to the AC-PC line, covering the whole brain and with a 1.5-mm slice thickness and a 0.94×0.94-mm pixel size, using the spoiled gradient echo sequence (TR = 10.3 ms; TE = 2.1 ms; FOV = 240×180 mm^2^; matrix = 256×192). All the MRI datasets were acquired on the same scanner (1.5T Signa Advantage Echospeed; General Electric), using the same acquisition protocol.

#### PET

Subjects also underwent a PET study within days of the MRI study. Data were acquired using an ECAT Exact HR+ scanner with isotropic resolution of 4.6×4.2×4.2 mm and axial field of view of 158 mm. Subjects were fasted for at least 4 hours before scanning. To minimize anxiety, the PET procedure was explained in detail beforehand. The head was positioned on a headrest relative to the canthomeatal line and gently restrained with straps. ^18^FDG uptake was measured in the resting condition, with eyes closed, in a quiet and dark environment. Subjects were told to avoid focusing on any specific mental process during scanning. A catheter was inserted into a vein of the arm to inject the radiotracer. Following ^68^Ga transmission scans, 3–5 mci of ^18^FDG were injected as a bolus at time 0, and a 10-min data acquisition period started 50 min post-injection. Sixty-three planes were acquired with septa out (3D acquisition), using a voxel size of 2.2×2.2×2.43 mm (x y z). During data acquisition, head motion was continuously monitored with, and whenever necessary corrected according to, laser beams projected onto ink marks drawn on the forehead.

### Image Processing

**Figure 4:**
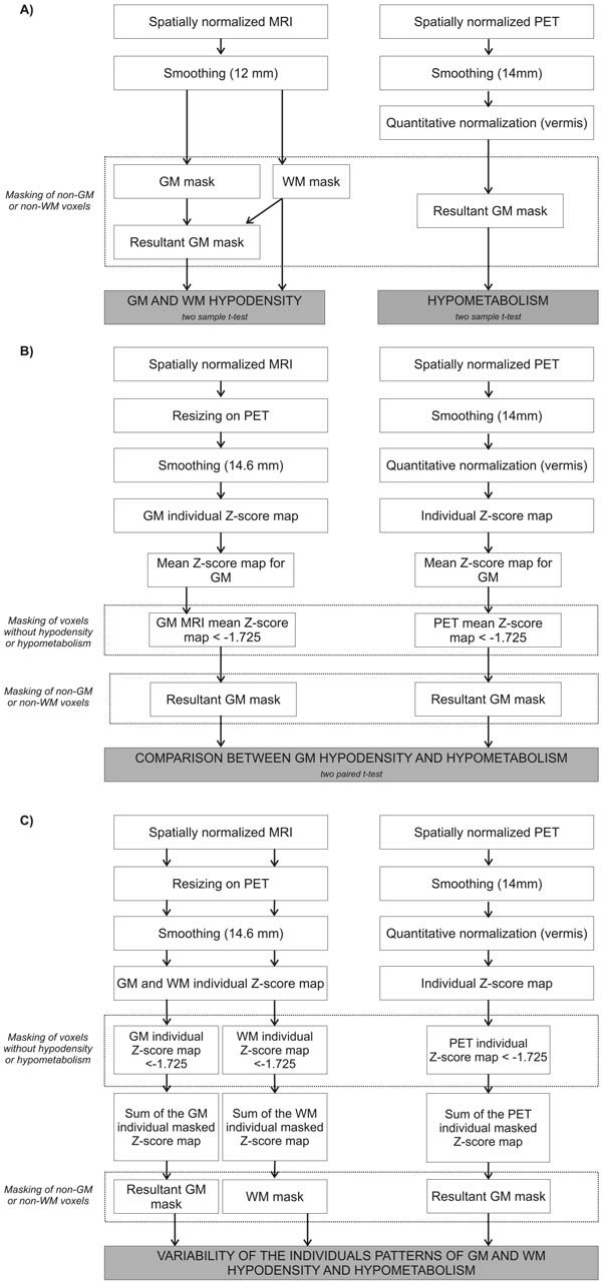
Schematic representation of the procedures for analyzing MRI and PET data after registration. A: Procedure for analyses of GM and WM hypodensity and GM hypometabolism; B: Procedure for the direct voxel-based comparison between morphological and metabolism abnormalities; C: Procedure for the analysis of the variability in the individual patterns of hypodensity and hypometabolism.

The data processing procedure was specifically designed notably to compare morphological and metabolic data, as previously described in detail [38], [52] and illustrated in Figure 4.

#### MRI

The MRI data were analyzed using VBM in SPM5, as described in detail elsewhere [70] and previously used in our laboratory [71], [72]. Briefly, the procedure included segmentation and registration (spatial normalization) of original MRI datasets using the default MNI template of SPM5 as priors (the use of a customized template is not recommended with SPM5 when small samples are used). Registered MRI data were then smoothed (see below for details).

#### PET

The PET data were first corrected for cerebrospinal fluid (CSF) and WM partial volume effects (PVE), using the voxel-by-voxel “modified Müller-Gartner” method [73], [74], described in detail elsewhere [75], and already used in our laboratory [38], [52], [71]. All image-processing steps for PVE correction were carried out using the PVE-lab software [75]. Using SPM5, the PVE-corrected PET datasets were then coregistered (rigid registration) onto their respective native MRIs and registered onto MNI, by reapplying the registration parameters estimated from the VBM protocol described above. Finally, after smoothing (see below for details), the resulting PET images were divided by their respective cerebellar vermis FDG uptake mean value (for the sake of quantitative normalization) to control for individual variations in the overall PET value [76]. The vermis was selected as a reference as it had the best-preserved FDG uptake value in our sample of KS patients relative to controls (data not shown).

### Smoothing

For the between-group comparison and in order to blur individual variations in gyral anatomy and increase the signal-to-noise ratio, the registered datasets were smoothed using a 12-mm Gaussian kernel. The registered PET datasets were also smoothed (14 mm) before quantitative normalization.

In order to directly compare morphological and metabolism GM abnormalities, a different Gaussian kernel was applied to the registered unsmoothed MRI datasets to compensate for the difference in the original spatial resolution between the two modalities [77], [78]. A 14.6-mm Gaussian kernel was used for the MRI GM data, resulting in an effective smoothness identical to PET images smoothed at 14 mm [79].

### Masking

The resulting MRI (GM and WM) and PET datasets were masked so as to include only GM or WM voxels of interest and to prevent any overlap between the two sets of voxels (see [52] for details). Briefly, mean images for the GM and WM partitions of the whole sample (n = 31) were created. The WM mask was created by thresholding the mean WM image above a value of 0.4. A preliminary GM mask was first obtained by thresholding the mean GM image above a value of 0.3, while the final GM mask was created by subtracting the WM mask from the preliminary GM mask. The resulting binary GM mask was applied to both the GM and PET datasets, and the binary WM mask to the WM data set.

### Z-Score Maps

The smoothed and masked GM, WM and PET images were used to create z-score maps [(patient individual value-control mean)/control standard deviation] for each patient and each modality.

### Statistical Analysis

Three complementary statistical analyses were performed: 1) between-group comparisons of GM density, WM density and FDG uptake (Figure 4A); 2) a within-group comparison between GM abnormalities and relative hypometabolism (Figure 4B); and 3) an analysis of variability in the individual patterns of GM and WM density abnormalities and relative hypometabolism across the KS group (Figure 4C).

#### Between-group comparisons

Group differences were assessed by generating maps of statistically significant GM and WM density abnormalities and hypometabolism in KS patients relative to controls in three independent analyses with the SPM5 two-sample t-test routine.

#### Within-group comparisons

Comparisons between the degrees of morphological and metabolism abnormalities were only performed for voxels with significant GM hypodensity or hypometabolism. To this end, mean z-score maps were first obtained by averaging the GM density and FDG uptake z-score maps for individuals. A mask image was then created by including only voxels with the mean MRI_(GM)_ and/or PET z-score <−1.725 (corresponding to the t-value for *p* (one-tailed) <0.05, with 20 degrees of freedom i.e. for a control sample size of 22). This mask was then applied to all individual z-score maps, and the degrees of GM hypodensity and hypometabolism were compared using the paired t-test in SPM5 with one group (KS) and two images per subject, i.e. the masked PET and MRI_(GM)_ z-score maps. Both contrasts were assessed (Z-PET<Z-MRI_(GM)_ and Z-MRI_(GM)_<Z-PET) to generate statistical maps reflecting predominant hypometabolism over GM hypodensity and vice-versa.

#### Variability of the individual patterns of abnormalities

To highlight interindividual variability in the patterns of GM and WM density abnormalities and GM hypometabolism in KS, we computed a map for each modality, displaying the number of patients with significant hypodensity or hypometabolism in each voxel. For this purpose, we first thresholded each individual GM, WM and PET z-score map below −1.725, in order to obtain a value of ‘1’ in voxels with significant change and a value of 0 elsewhere for each patient’s map. We then added the masked individual z-score maps for GM, WM and PET images separately. The three resulting images reflected the frequency (from 0 to 9) in each voxel of significant GM or WM abnormalities or hypometabolism in our sample. An invariant pattern, which could be considered as a brain biomarker, was operationally defined here as involving all 9 KS patients.

### Statistical Threshold and Display of Results

SPM-T maps of GM and WM damage and hypometabolism were thresholded using an FDR-corrected *p*-value of <0.001, with a minimum cluster size of 200 voxels. This stringent threshold was selected because lower thresholds highlighted abnormalities across the whole brain. When relevant, the results were reported using a less stringent FDR-corrected *p*-value cut-off of <0.005. An FDR-corrected *p*-value of <0.01 with a minimum cluster size of 200 voxels was used for the comparison between GM density abnormalities and hypometabolism. Anatomical localization was based on the superimposition of SPM-T maps onto the MNI template using MRIcro [80], [81].
